# Unveiling the Dynamics of Antimicrobial Resistance: A Year-Long Surveillance (2023) at the Largest Infectious Disease Profile Hospital in Western Romania

**DOI:** 10.3390/antibiotics13121130

**Published:** 2024-11-25

**Authors:** Sorina Maria Denisa Laitin, Luminita Mirela Baditoiu, Ruxandra Laza, Irina-Maria Stefan, Razvan Sebastian Besliu, Septimiu Radu Susa, Cristian Oancea, Emil Robert Stoicescu, Diana Manolescu, Corneluta Fira-Mladinescu

**Affiliations:** 1 Epidemiology University Clinic, Department XIII, “Victor Babes” University of Medicine and Pharmacy Timisoara, Eftimie Murgu Square No. 2, 300041 Timisoara, Romania; laitin.sorina@umft.ro (S.M.D.L.); baditoiu.luminita@umft.ro (L.M.B.); 2 Clinical Hospital of Infectious Diseases and Pneumoftiziology ‘Dr. Victor Babes’ Timisoara, 300310 Timisoara, Romania; laza.ruxandra@umft.ro; 3 Infectious Diseases University Clinic, Department XIII, “Victor Babes” University of Medicine and Pharmacy Timisoara, 2 Eftimie Murgu Square, 300041 Timisoara, Romania; 4 Regional Center for Public Health Timisoara, 300226 Timisoara, Romania; irina.stefan@umft.ro; 5 Doctoral School, “Victor Babes” University of Medicine and Pharmacy Timisoara, Eftimie Murgu Square 2, 300041 Timisoara, Romania; septimiu.susa@umft.ro; 6 Epidemiology Clinic, “Pius Brinzeu” Emergency Clinical County Hospital Timisoara, Liviu Rebreanu Boulevard No. 156, 300723 Timisoara, Romania; besliusebastian@gmail.com; 7 Pneumology University Clinic, Department XIII, “Victor Babes” University of Medicine and Pharmacy Timisoara, Eftimie Murgu Square, Nr. 2, 300041 Timisoara, Romania; oancea@umft.ro; 8 Center for Research and Innovation in Precision Medicine of Respiratory Diseases (CRIPMRD), “Victor Babes” University of Medicine and Pharmacy Timisoara, Eftimie Murgu Square, Nr. 2, 300041 Timisoara, Romania; dmanolescu@umft.ro; 9 Radiology and Medical Imaging University Clinic, Department XV, “Victor Babes” University of Medicine and Pharmacy Timisoara, Eftimie Murgu Square No. 2, 300041 Timisoara, Romania; 10 Research Center for Pharmaco-Toxicological Evaluations, “Victor Babes” University of Medicine and Pharmacy Timisoara, Eftimie Murgu Square No. 2, 300041 Timisoara, Romania; 11 Field of Applied Engineering Sciences, Specialization Statistical Methods and Techniques in Health and Clinical Research, Faculty of Mechanics, “Politehnica” University Timisoara, Mihai Viteazul Boulevard No. 1, 300222 Timisoara, Romania; 12 Hygiene Division, Department of Microbiology, “Victor Babes” University of Medicine and Pharmacy Timisoara, Victor Babes No. 16, 300226 Timisoara, Romania; fira-mladinescu.corneluta@umft.ro; 13 Center for Study in Preventive Medicine, “Victor Babes” University of Medicine and Pharmacy Timisoara, Eftimie Murgu Square No. 2, 300041 Timisoara, Romania

**Keywords:** antimicrobial resistance, multi-drug resistant bacteria, bacterial culture analysis, infection control measures, antimicrobial stewardship

## Abstract

**Background/Objectives***:* Antimicrobial resistance (AMR) is a critical global health threat, leading to increased morbidity, mortality, and healthcare costs. This study aimed to identify the most common bacterial pathogens and their resistance profiles from 2179 positive clinical cultures from inpatients at “Victor Babes” Hospital of Infectious Disease and Pneumoftiziology Timisoara in 2023. **Methods***:* Samples were collected from sputum, bronchial aspiration, hemoculture, urine, wound secretions, catheter samples, and other clinical specimens. **Results***:* Key pathogens identified included *Klebsiella pneumoniae*, *Escherichia coli*, *Staphylococcus aureus*, *Pseudomonas aeruginosa*, and *Acinetobacter baumannii*, with notable resistance patterns, observed *K. pneumoniae* exhibited high resistance rates, notably 41.41% in Quarter 1, while *E. coli* showed 35.93% resistance in the same period. *S. aureus*, particularly MRSA, remained a persistent challenge, with 169 cases recorded over the year. *A. baumannii* and *P. aeruginosa* displayed alarming levels of multi-drug resistance, especially in Quarter 3 (88.24% and 22.02%, respectively). Although there was a general decline in resistance rates by Quarter 4, critical pathogens such as *S. aureus* and *K. pneumoniae* continued to exhibit significant resistance (81.25% and 21.74%, respectively). **Conclusions***:* The study’s findings align with the broader antimicrobial resistance trends observed in Romania, where high resistance rates in *K. pneumoniae*, *E. coli*, *S. aureus* (MRSA), *Acinetobacter*, and *Pseudomonas* species have been widely reported, reflecting the country’s ongoing struggle with multi-drug-resistant infections. Despite some reductions in resistance rates across quarters, the persistent presence of these resistant strains underscores the critical need for strengthened antimicrobial stewardship, infection control measures, and continuous surveillance to combat the growing threat of AMR in Romania and similar healthcare settings.

## 1. Introduction

Antimicrobial resistance (AMR) is a growing global public health challenge, posing significant threats to the effective treatment of infectious diseases [1,2]. The increasing prevalence of multi-drug resistant (MDR) bacterial strains has led to higher morbidity, mortality, and healthcare costs worldwide [3,4]. The World Health Organization (WHO) has identified AMR as one of the top ten global public health threats facing humanity [5]. Understanding the local epidemiology of AMR is crucial for developing targeted strategies to mitigate its impact [6,7,8].

In recent years, numerous studies have highlighted the alarming rise in antibiotic-resistant bacteria across different regions [1,6,7,9]. For instance, a study conducted by Beig et al. reported high resistance rates in *Enterobacteriaceae* and *Staphylococcus* species, emphasizing the need for continuous surveillance and adaptive interventions [10,11]. Similarly, research published by Niu et al. and Wu et al. identified significant resistance in *Acinetobacter* species, correlating with increased healthcare-associated infections and prolonged hospital stays [12,13,14].

AMR poses a significant challenge across various pathologies, affecting the treatment efficacy of infections ranging from urinary tract infections (UTIs) to tuberculosis (TB), especially in different conditions like diabetes [9,15,16,17,18]. In UTIs, resistance to commonly used antibiotics like ciprofloxacin and trimethoprim-sulfamethoxazole is increasing, complicating management and often requiring the use of more potent, broad-spectrum antibiotics [9,16,17]. Respiratory infections, such as community-acquired pneumonia, are seeing rising resistance rates in pathogens like *Streptococcus pneumoniae* to penicillin and macrolides, leading to higher morbidity and mortality rates [18,19,20]. Methicillin-resistant *Staphylococcus aureus* (MRSA) continues to be a critical concern in both hospital and community settings, necessitating alternative treatments like vancomycin [21]. Multidrug-resistant TB (MDR-TB) has emerged as a significant public health threat, requiring prolonged treatment with second-line drugs that have more severe side effects [18]. AMR not only undermines the effectiveness of standard treatments but also increases the burden on healthcare systems, emphasizing the need for ongoing surveillance, prudent antibiotic use, and the development of novel antimicrobial agents [1,11,17].

Romania, like other countries in Eastern Europe, faces significant challenges in addressing AMR. According to recent reports from the WHO and the European Centre for Disease Prevention and Control (ECDC), Romania reports high levels of resistance to several key antibiotics. For example, carbapenem resistance is notably high in Romania. This resistance trend, particularly for bacteria such as *E. coli* and *K. pneumoniae*, presents a serious challenge to public health. Data from 2021 also highlighted Romania’s struggle with resistance to other bacteria like MRSA and *P. aeruginosa*, posing significant risks to patient safety, especially in healthcare settings where infections are more likely to occur. This is consistent with a broader trend of higher AMR rates observed in Eastern European countries compared to their Western European counterparts [22]. These improvements underline the importance of Romania’s efforts in improving antimicrobial stewardship, infection prevention, and control practices to mitigate the spread and impact of resistant infections. Strengthening AMR surveillance and fostering public health interventions are crucial steps Romania must take to combat this growing health threat.

This study aimed to identify the most common bacteria in various clinical cultures and their resistance profiles from inpatients at “Victor Babes” Hospital of Infectious Disease and Pneumoftiziology Timisoara over the course of 2023. By analyzing the prevalence and resistance patterns of bacterial pathogens, this study provides valuable insights into the local AMR landscape and may help inform healthcare practices and policy decisions.

## 2. Results

### 2.1. Patient and Isolate Characteristics

**Figure 1:**
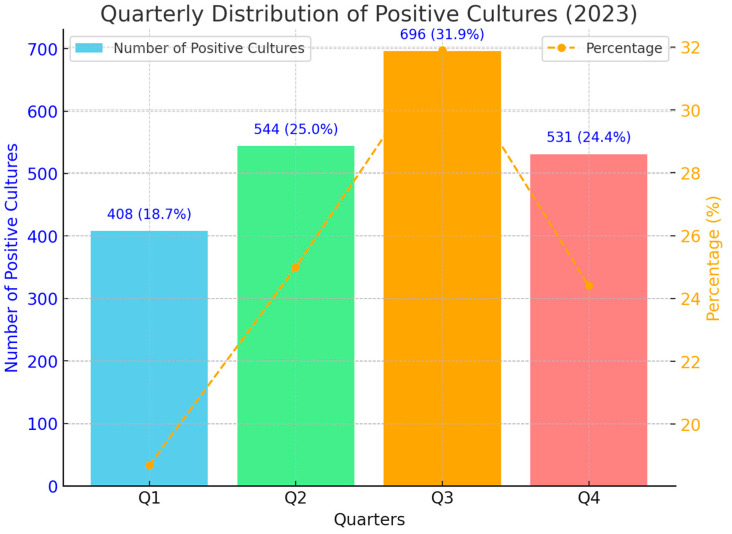
The distribution’s histogram during quarters in 2023.

In 2023, a total of 2179 positive cultures were identified in our hospital. The quarterly distribution of these cultures shows a notable variation throughout the year. In the first quarter, there were 408 positive cultures, accounting for 18.7% of the yearly total. This number increased in the second quarter to 544 positive cultures, representing 25% of the annual total. The highest number of positive cultures occurred in the third quarter, with 696 cases, comprising 31.9% of the total. In the fourth quarter, the number of positive cultures decreased to 531, making up 24.4% of the yearly total. The distribution’s histogram during quarters in 2023 is presented in Figure 1.

The data provided in the table below reflects the distribution of various sample types collected for diagnostic purposes, with a total of 2179 samples analyzed. The highest percentage of samples comes from sputum, accounting for 30.1% of the total, with 656 samples.

Following sputum, bronchial aspiration samples represent the second largest category, comprising 22.6% of the total with 493 samples.

Urine cultures come next with 284 samples, making up 13.0% of the total.

Wound secretion samples, which include a variety of body fluids, account for 18.6% of 407 samples. This category included wound exudates and other body secretions.

Hemocultures are used to detect infections in the blood and account for 7.4% of the total, with 163 samples. Catheter-related samples comprise 5.6% of the total, with 123 samples.

Lastly, the other category includes 53 samples, making up 2.4% of the total. This category might consist of less common or unspecified sample types.

**Figure 2:**
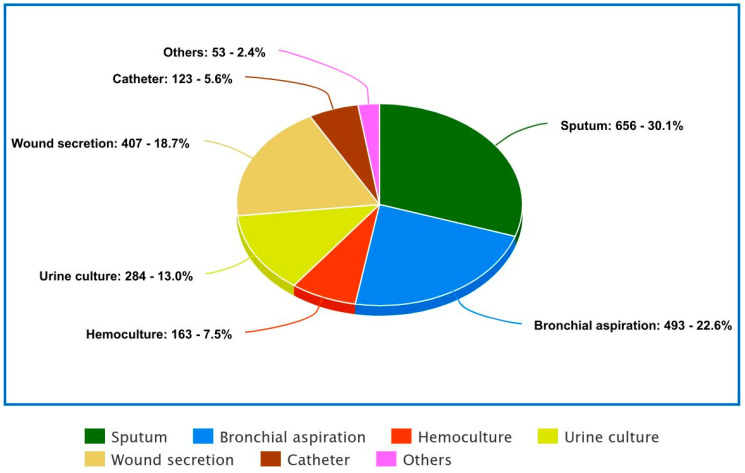
Number and percentage of isolates (%) over the surveillance period (Timisoara Hospital, 2023) by specimen type.

In the first quarter, bronchial aspiration was the most frequently used positive sample, but in the second, third, and fourth quarters, the positive samples obtained from sputum were the most common ones. Furthermore, a pie chart of the positive samples’ distribution during quarters in 2023 is presented in Figure 2. The distribution of positive samples during quarters is presented in Table 1.

### 2.2. Antimicrobial Susceptibility Testing Results Around Quarters

In Q1, analyzing 408 positive samples, a significant proportion, 176 strains (43.13%), exhibited antibiotic resistance. These resistant strains include MRSA, MRSE, carbapenem and cephalosporin-resistant *Enterobacteriaceae*, ESBL-producing strains with extended resistance phenotypes, and VRE. Predominant findings in Q1 were *Enterobacteriaceae* represented the most frequently identified family, accounting for 167 out of 408 samples (40.93%). Among these, 41 out of 99 *K. pneumoniae* (41.41%) showed resistance to carbapenems and/or cephalosporins and/or beta-lactams. Similarly, 23 out of 64 *E. coli* strains (35.93%) were resistant to cephalosporins and/or beta-lactams. The *Staphylococcus* group was the second most common group, with 91 out of 408 samples (22.3%). Of these, 50 out of 91 strains (54.9%) displayed resistance to methicillin (MRSA 36.2% and MRSE 18.6%). *P. aeruginosa* ranked third in frequency, with 58 out of 408 samples (14.21%). Within this group, 12 out of 58 strains (20.68%) were resistant to carbapenems and/or cephalosporins and/or beta-lactams.

In Q2, from 544 positive samples, 205 strains (37.68%) demonstrated antibiotic resistance, including MRSA, MRSE, carbapenem and cephalosporin-resistant *Enterobacteriaceae*, and ESBL-producing strains with extended resistance phenotypes. Notably, no VRE strains were identified. Predominant findings in Q2 were that *Enterobacteriaceae* was the most frequently identified family, with 87 species of demonstrated antibiotic resistance out of 544 samples (15.99%). Among these, 55 out of 146 *K. pneumoniae* (37.67%) exhibited resistance to carbapenems and/or cephalosporins and/or beta-lactams. Similarly, 22 out of 111 *E. coli* strains (19.81%) showed resistance to carbapenems and/or cephalosporins and/or beta-lactams. The *Staphylococcus* group ranked second in frequency with 116 out of 544 samples (21.3%). Of these, 57 out of 117 strains (49.13%) demonstrated high resistance to methicillin (MRSA and MRSE). *P. aeruginosa* was the third most common group in antibiotic resistance, with 21 out of 92 samples (22.82%). Within this group, 12 out of 76 *Pseudomonas* strains (15.78%) were resistant to carbapenems and/or cephalosporins and/or beta-lactams, and 9 out of 16 *Acinetobacter* strains (56.25%) showed resistance to these antibiotics. A peak in multi-drug resistant *K. pneumoniae* was reported in urinary cultures during Q2 2023, with 26 out of 29 samples (89.65%) affected.

In Q3, a total of 696 bacterial strains were identified from various biological samples collected from patients in the hospital. Among these, 312 strains exhibited resistance, representing 44.82% of the total samples.

Findings in Q3 were the following: *Enterobacteriaceae* was the most frequently identified family with 338 strains. Of these, 126 strains (37.27%) exhibited resistance to multiple antibiotics (carbapenems, cephalosporins, beta-lactam beta-lactamase inhibitors combination, macrolides, lincosamides, fluoroquinolones). *K. pneumoniae* was demonstrated in 166 strains identified, with 71 showing MDR phenotypes (42.77%). Notably, 34 of these were classified as XDR (47.88% of MDR *K*. *pneumoniae*). *E. coli* with 107 strains was identified, with 38 exhibiting MDR phenotypes (35.51%). Among these, 9 strains were ESBL-producing *E. coli* (23.68% of MDR *E. coli*)*. Proteus* spp. with 26 strains was identified, with 6 showing MDR phenotypes (23.08%). *Enterobacter* spp. with 18 strains was identified, with 7 showing MDR phenotypes (38.89%). *Staphylococcaceae* was the second most common family, with 178 strains. From these, 105 strains (58.98%) exhibited resistance to multiple antibiotics (beta-lactam antibiotics, macrolides, fluoroquinolones, cephalosporins), with MRSA and MRSE with 41.1%. *S. aureus*: 139 strains were identified, with 85 showing MDR phenotypes (61.15%). Among these, 62 strains were MRSA (44.6% from *Staphylococcus*), and 34 were Macrolide-lincosamide-streptogramin B (MLSB), counting 40% of MDR *S*. *aureus*. Additionally, 48 strains were identified as MSSA (26.96%). *S. epidermidis*: 29 strains were identified, with 17 showing MDR phenotypes (58.62%). Among these, 11 strains were MRSE (64.70% of MDR *S*. *epidermidis*), and 3 were MLSB (17.64% of MDR *S*. *epidermidis*). *Pseudomonas* was third in frequency with 109 strains with the following resistance profile: 24 strains (22.02%) exhibited resistance to multiple antibiotics (cephalosporins, fluoroquinolones, macrolides, carbapenems, lincosamides, beta-lactamase inhibitors). *Acinetobacter* was fourth in frequency with 34 strains with the following resistance profile: 30 strains (88.24%) exhibited resistance to multiple antibiotics (cephalosporins, fluoroquinolones, macrolides, carbapenems, lincosamides, beta-lactamase inhibitors). *Enterococcus* was fifth in frequency with 18 strains with the following resistance profile: 7 strains (47.06%) exhibited resistance to multiple antibiotics (glycopeptides, fluoroquinolones, penicillins). Among these, 3 strains were identified as VRE (42.85% of MDR *Enterococcus*). Other Genera (e.g., *Providencia*, *Bacillus*, *Stenotrophomonas maltophilia*, *Streptococcus*) with a prevalence of 63 strains identified with the following resistance profile: 29 strains (46.03%) exhibited resistance to multiple antibiotics (cephalosporins, macrolides, lincosamides, fluoroquinolones, beta-lactamase inhibitors, penicillins).

In Q4 of 2023, a total of 531 bacterial strains were identified from various biological samples collected from patients in the hospital. Among these, 156 strains exhibited resistance, representing 29.37% of the total samples. *Enterobacteriaceae* predominance was demonstrated, with *K. pneumoniae* (23.33%) and *E. coli* (20.25%) showing notable resistance rates, including XDR and ESBL phenotypes. *Staphylococcaceae* resistance was proven with a prevalence of MRSA (42/135, 31.1%) and MRSE (22/135, 16.2%) phenotypes, particularly among *S. aureus* and *S. epidermidis*. *Pseudomonas* and *Acinetobacter* resistance demonstrated the following rates in carbapenem-resistant *P. aeruginosa* (11/90, 12.2%) and *A. baumannii* (14/32, 43.7%). *Enterococcus* resistance demonstrated a percentage of 53.33% VRE+ and HLAR+. Over the year, there is fluctuation in the percentage of resistant strains, with the highest resistance observed in Q3 (44.8%) and the lowest in Q4 (29.3%). The trend could be seen as cyclical, with a decrease from Q1(43.1%) to Q2 (37.6%) to Q2, an increase in Q3, and a marked decrease in Q4. While Q3 represents the peak of resistance, the sharp decline in Q4 is visible. The Mantel-Haenszel test for trend results in a test statistic of 298.7 with a *p*-value < 0.001.

**Figure 3:**
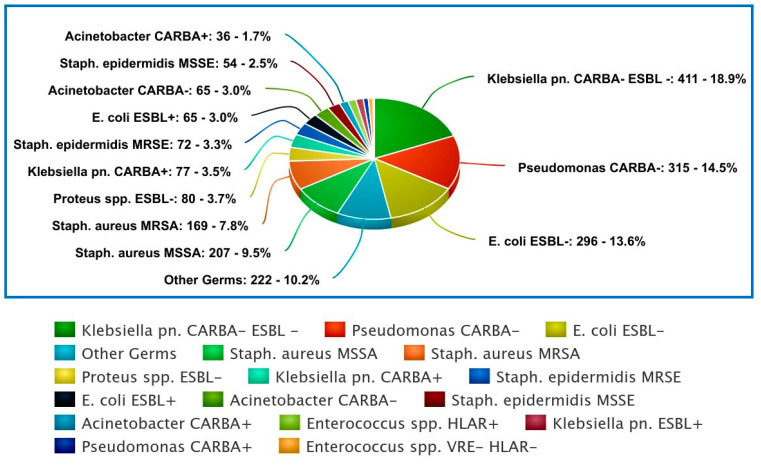
Number (n) and percentage (%) of bacterial infections during the surveillance period (2023) by organism and resistance phenotype.

Table S1 (Supplementary Files) provides a detailed breakdown of different bacterial infections across various sections (units) of the hospital. It categorizes the infections based on the type of bacteria and their resistance patterns. The data is organized in a manner that shows the number of infections for each category within each section. Furthermore, Figure 3 presents the prevalence of bacterial infections during 2023.

The data from Table S1 highlights several key points regarding bacterial infections across different sections of the healthcare facility. The ID 1 unit has a notably high number of infections, with 74 cases of *E. coli* ESBL- and 37 cases of *Pseudomonas* CARBA-. In contrast, ID 2 shows relatively low infection counts across all bacterial categories. The PN 1 unit reports a significant number of 140 *K. pneumoniae*. CARBA- ESBL- infections, while PN 2 also records a high count of 145 *K. pneumoniae* CARBA- ESBL- infections and 109 cases of *Pseudomonas* CARBA-. The ICU has notable infection numbers, with 48 cases of Staph. aureus MRSA and 28 cases of *K. pneumoniae* CARBA+. The RMRU, TOR. S. and ID 2 generally exhibit lower overall infection counts compared to other sections.

The quarterly resistance rates (%R) for bacteria species are presented in the Supplementary Files: Figure S1—Quarterly resistance rates for Enterobacterales, Figure S2—Quarterly resistance rates for Gram-positives, Figure S3—Quarterly resistance rates for Non-Fermenters. Furthermore, Table S2 presents the number of isolates and %MDR and %XDR in 2023.

### 2.3. MRSA and MRSE Around Quarters

**Figure 4:**
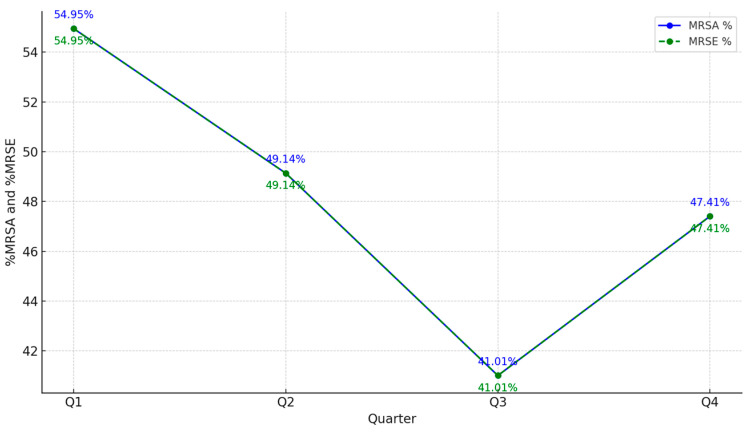
%MRSA and %MRSE trends across the quarters in 2023.

The Mantel-Haenszel Test for trend applied to the MRSA and MRSE data across the four quarters yielded a *p*-value of 0.165. Therefore, the fluctuations observed over time are represented in the figure below (Figure 4).

## 3. Discussion

In analyzing the resistance patterns of various bacterial strains across four quarters, we observe distinct trends and fluctuations that underscore the dynamic nature of antibiotic resistance.

The quarterly analysis of resistance patterns highlights distinct trends, with the highest levels observed in Quarter 3, particularly in *Acinetobacter* (88.24%) and *Pseudomonas* (22.02%). This alarming rise underscores an urgent need for intensified infection control and antimicrobial stewardship measures. Notably, the peak during this period may correlate with seasonal factors, including elevated temperatures and increased patient mobility, which can contribute to higher resistance rates [11]. Such high resistance levels often lead to prolonged hospital stays and an increased burden on healthcare resources [23,24].

Persistently high resistance rates in *K. pneumoniae* and *S. aureus* across all quarters, despite some reduction in Quarter 4, reflect the substantial challenges in managing these infections in hospitalized patients. Carbapenemase-producing and ESBL-producing *K. pneumoniae*, coupled with the prevalence of methicillin-resistant *S. aureus* (MRSA), continue to complicate treatment options. These findings are similar to other recent findings [10,21,25]. These pathogens underscore the critical need for stringent infection control practices and the development of novel therapeutic approaches to counteract their high resistance levels effectively [22,23].

The reduction in resistance seen in Quarter 4, particularly in *K. pneumoniae* and *S. aureus*, may signify the positive influence of targeted antimicrobial stewardship programs implemented after Q3’s results. Such adaptive interventions appear essential to mitigate resistance trends and sustain effective infection management. This observed decline suggests that continued stewardship efforts, adapted to the resistance patterns and healthcare setting demands, can meaningfully curb resistance among these pathogens [26,27].

The findings align with broader regional and national trends in Romania and Eastern Europe, where high resistance rates in *K. pneumoniae*, *E. coli*, *S. aureus*, and *Acinetobacter* represent ongoing public health concerns [22,28]. These pathogens are central to multi-drug resistance issues in the region, echoing similar data reported in national surveillance [26,27].

In analyzing the resistance patterns of various bacterial strains across four quarters, we observed trends that align with those reported in similar studies. For instance, Majumder et al. and Iredell et al. also found high resistance rates in *K. pneumoniae* and *E. coli*, especially in ICU and pulmonary units. This mirrors our finding of high resistance in *K. pneumoniae* (41.41%) and *E. coli* (35.93%) in Quarter 1. However, our data in Q4 showed a reduction in *K. pneumoniae* resistance to 21.74%, a trend not observed in other studies like Poirel et al., who reported a consistent rise in resistance across the year. The discrepancy could be attributed to local antimicrobial stewardship programs or regional differences in patient demographics and clinical practices [23,29,30].

Comparing these findings with similar studies, we see parallel trends in antimicrobial resistance. For instance, a study published by Beig et al. reported high resistance rates in Enterobacteriaceae and Staphylococcus, aligning with the trends observed in our study [10,17]. Another study published by Niu et al. and Wu et al. highlighted the significant resistance in *Acinetobacter*, corroborating the alarming rates seen in Q3 of our analysis [13,14]. Furthermore, a recent study published by De Blasiis et al. noted the critical impact of *A. baumannii* in critical care units globally, necessitating specialized infection control protocols [31]. Lu et al. highlighted that increased travel during vacation periods could facilitate the spread of resistant strains. Additionally, the elevated temperatures in Q3 may contribute to higher bacterial colonization rates in asymptomatic carriers, as noted by Bengtsson-Palme et al. in their analysis of seasonal AMR trends [32,33]. These environmental factors, combined with higher rates of hospital admissions during the summer, could explain the spike in resistance [34,35,36].

The results of year-long surveillance revealed significant antimicrobial resistance patterns across various bacterial pathogens in 2023, echoing global trends [5,7,34]. *S. aureus* exhibited high resistance rates, with 169 cases (44.9%) of MRSA notably concentrated in the Pulmonology (PN) and Intensive Care Unit (ICU) sections. The results of the Mantel-Haenszel Test for Trend, with a *p*-value of 0.165, indicate that the changes in MRSA and MRSE rates across the four quarters of 2023 are not statistically significant. In other words, although fluctuations in MRSA and MRSE percentages are observed from quarter to quarter, they do not represent a consistent increasing or decreasing trend over time. This suggests that the variations in MRSA and MRSE rates could be due to random fluctuations or external factors rather than a significant underlying trend in the data. From a clinical perspective, this reinforces the importance of continuous monitoring of MRSA and MRSE rates to detect any meaningful changes over longer periods or to identify other contributing factors, such as seasonal influences, changes in infection control practices, or variations in patient demographics. However, this persistent high level of MRSA is consistent with reports from Shoaib et al., which document similar MRSA prevalence in hospital settings, highlighting the need for robust infection control measures and targeted antibiotic stewardship programs [12,37]. *S. epidermidis*, both methicillin-sensitive and methicillin-resistant, showed lower but still significant incidences, particularly in the PN and ICU units, aligning with literature that indicates coagulase-negative staphylococci are significant contributors to nosocomial infections [5,7,34,36].

The study also identified high resistance rates in *Enterobacteriaceae*, particularly *K. pneumoniae* and *E. coli.* Non-carbapenemase-producing, non-ESBL-producing *K. pneumoniae* (411 cases) were predominant in PN units, with carbapenemase-producing strains (77 cases) also significant, reflecting findings from the Niu et al. and Wu et al. that highlight increasing carbapenem resistance in *Enterobacteriaceae* [13,14]. ESBL-producing *E. coli* strains (65 cases) were notably present in Infectious Disease and PN units, consistent with global challenges posed by ESBL-producing *E. coli*, as discussed in the above-mentioned articles. Furthermore, *A. baumannii* and *P. aeruginosa* exhibited high resistance, with 315 carbapenem-resistant *Acinetobacter* cases predominantly in PN units, aligning with similar reports of *Acinetobacter* as a leading cause of hospital-acquired infections [12,13,14]. *Pseudomonas* strains, particularly carbapenem-resistant variants (59 cases), were prevalent in ICU and PN units, corroborating findings from studies that underscore the threat of *Pseudomonas* in critical care settings [38,39]. These patterns emphasize the urgent need for comprehensive AMR surveillance and adaptive antibiotic policies to manage and mitigate resistance effectively [40,41].

Our study’s findings align closely with the broader AMR trends observed in Romania and Eastern Europe, where high resistance rates in key pathogens such as *K. pneumoniae, E. coli*, *S. aureus* (MRSA), and *P. aeruginosa* have been widely reported. In line with national data from the WHO and ECDC, we observed significant carbapenem resistance in *K. pneumoniae*, particularly in Quarter 3, reflecting Romania’s ongoing challenge in managing multidrug-resistant infections [22]. Similarly, *E. coli* showed resistance rates of 35.93% (Q1), mirroring high national resistance levels to third-generation cephalosporins and fluoroquinolones. Our study also highlighted 169 cases of MRSA, concentrated in Pulmonology and ICU units, consistent with Romania’s high MRSA prevalence compared to Western Europe. *P. aeruginosa* presented the highest resistance rates of 22.02% in Quarter 3, echoing national concerns over healthcare-associated infections. These findings reflect the broader trend of higher AMR rates in Eastern Europe, driven by factors such as limited antimicrobial stewardship and higher rates of hospital-acquired infections. Strengthening surveillance, infection control, and stewardship programs are crucial steps for Romania to combat AMR effectively [22].

This study has several limitations that must be acknowledged. First, the retrospective design may introduce selection bias, as only patients who had samples sent for culture were included, potentially skewing the resistance rates observed. The infection counts presented reflect the number of positive cultures identified during the study period. However, the absence of baseline data on the total number of culture requests limits our ability to accurately determine culture-positive rates or compare infection rates across different wards or quarters. Additionally, its focus on a single specialized hospital in Western Romania, dealing mainly with respiratory pathology and infectious diseases, limits the representativeness of the findings across the region’s broader healthcare landscape. These limitations suggest the need for a cautious interpretation of the findings and highlight areas for improvement in future studies.

Future research should prioritize prospective studies, advanced molecular diagnostic techniques, and collaborative efforts to enhance the accuracy and applicability of resistance data. Continuous monitoring and adaptive management practices are crucial to mitigate the evolving threat of antimicrobial resistance, ensuring effective treatment protocols and improved patient outcomes.

## 4. Materials and Methods

### 4.1. Study Design and Setting

This retrospective study was conducted at “Victor Babes” Hospital of Infectious Disease and Pneumoftiziology Timisoara, the largest hospital of infectious disease and pneumology from the West Development Region of Romania, with a population of 1.66 million [42].

The duration of the study was twelve months, specifically in the year 2023, from 1 January to 31 December 2023. The total number of admitted patients throughout this period was 6440. Of them, 290 were admitted to the ICU. The primary objective was to identify the most common and clinically relevant bacteria found in various clinical cultures from inpatients, including sputum, bronchial aspiration, hemoculture, urine culture, wound secretion, catheter samples, and other relevant specimens.

### 4.2. Inclusion Criteria

The study included only inpatients who were admitted to the “Victor Babes” Hospital of Infectious Disease and Pneumoftiziology Timisoara during the study period from 1 January to 31 December 2023.

Only clinical samples that yielded positive bacterial cultures were included in the analysis. This ensures that only confirmed bacterial infections or superinfections were evaluated. Samples with negative cultures or without significant bacterial growth were excluded from the study.

Samples collected from a variety of clinical sources, including sputum, bronchial aspirations, hemocultures, urine cultures, wound secretions, catheter samples, and other relevant specimens (e.g., cerebrospinal fluid, pleural fluid), were considered for inclusion, provided they tested positive for bacterial pathogens.

In cases where multiple positive samples were obtained from the same patient, only the first isolate per patient was included in the analysis, following CLSI guidelines, to prevent duplicate data from influencing the results.

### 4.3. Sample Collection

Clinical samples were collected from hospitalized patients with suspected bacterial infections or superinfections during their stay. Collected specimens included sputum, bronchial aspirations, hemocultures, urine cultures, wound secretions, catheter samples (including bronchial, central-line catheter, peripheral-line catheter, and arterial), and other clinical specimens like cerebrospinal or pleural fluid. Each sample was collected in a sterile container and transported to the laboratory within two hours to preserve specimen quality and prevent contamination.

### 4.4. Bacterial Identification

Bacterial identification focused on specific pathogens, including *S. aureus* (MSSA, MRSA), *S. epidermidis* (MSSE, MRSE), *K. pneumoniae*, *E. coli*, *Proteus*, *Acinetobacter*, *Pseudomonas*, and *Enterococcus* species. Additionally, any other unlisted bacteria were classified as “other germs” for comprehensive coverage.

MDR bacteria are defined as having acquired resistance to at least one agent in three or more antimicrobial categories. XDR (extensively drug-resistant) bacteria are resistant to all but one or two categories, meaning they remain susceptible to only one or two antimicrobial categories [43].

### 4.5. Laboratory Methods 

The laboratory was reaccredited by RENAR in December 2020, holding the accreditation certificate LM 1198, which specifies that the laboratory meets the requirements of SR EN ISO 15189:2013 [44] and is competent to perform medical analyses.

#### 4.5.1. Sample Processing

Samples were collected in sterile containers and transported to the laboratory within 2 h of collection.

Hemoculture samples were processed using automated blood culture systems BD (Becton, Dickinson and Company) Bactec™ (Macquarie Park, Australia).

#### 4.5.2. Culture and Identification

Firstly, samples were inoculated on appropriate media (e.g., Columbia blood agar base, blood agar, MacConkey agar from Oxoid™, Basingstoke, UK) and incubated at 37 °C for 24–48 h.

Bacteria were identified using standard microbiological techniques, including Gram staining, colony morphology, and biochemical tests (e.g., catalase, coagulase).

Furthermore, automated technologies such as VITEK 2 (bioMérieux, Marcy-l’Étoile, France) were employed for bacterial identification and antibiotic susceptibility testing in specific instances, including samples from patients in ICU, sepsis, or with meningitis.

#### 4.5.3. Antimicrobial Susceptibility Testing

Antimicrobial susceptibility was determined using the Kirby-Bauer disk diffusion method following CLSI guidelines [43].

Minimum Inhibitory Concentration (MIC) values for selected antibiotics were determined using automated systems or E-test strips.

Specific tests for detecting methicillin resistance (PBP2a latex agglutination for MRSA/MRSE), ESBL production (double-disk synergy test), and carbapenemase production (modified Hodge test, Carba NP test) were performed.

### 4.6. Data Collection and Analysis

#### 4.6.1. Data Recording

Data on the type of sample, bacterial species, and antimicrobial resistance profile were recorded for each patient.

As recommended by the CLSI guidelines, only the first isolate per patient was considered for analysis, ensuring that no multiple isolates from the same patient were included [45].

#### 4.6.2. Statistical Analysis

The statistical analysis was conducted using MedCalc^®^ Statistical Software version 22.030, developed by MedCalc^®^ Software Ltd. in Ostend, Belgium. The software is accessible at the URL https://www.medcalc.org and was accessed on 15 June 2024.

The collected data were meticulously recorded in a secure computerized database using Microsoft^®^ Excel^®^ for Microsoft 365 MSO (Version 2406 Build 16.0.17726.20078) 64-bit, which was launched on 11 June 2024.

Descriptive statistics were used to summarize the frequency of different bacterial species and their resistance patterns.

### 4.7. Ethical Considerations

The study was conducted following the ethical principles outlined in the Declaration of Helsinki.

Patient confidentiality was maintained by anonymizing all patient identifiers in the dataset.

The study protocol was reviewed and approved by the Institutional Ethics Committee of “Victor Babes” Hospital of Infectious Disease and Pneumoftiziology Timisoara (approval number 6959/2024).

## 5. Conclusions

This study provides a comprehensive analysis of antimicrobial resistance patterns at “Victor Babes” Hospital in Timisoara over a one-year period (2023). The findings reveal significant resistance rates among key bacterial pathogens, with notable fluctuations across different quarters. *Enterobacteriaceae*, particularly *K. pneumoniae* and *E. coli*, consistently exhibited high resistance levels, while *S. aureus* showed persistently high methicillin resistance. The alarming rates of multi-drug resistance in *Acinetobacter* and *Pseudomonas* species further underscore the urgent need for robust antimicrobial stewardship and infection control measures.

Despite a general trend towards decreasing resistance in some quarters, the high prevalence of resistant strains, especially in clinically significant pathogens, highlights the ongoing challenge of managing bacterial infections in a healthcare setting. All in all, this study contributes valuable insights to the global discourse on antibiotic resistance and underscores the critical need for sustained vigilance and innovation in combating this public health challenge.

## Figures and Tables

**Table 1:** Number (n) and percentage (%) of isolates by quarter and specimen type.

Sample	Quarter 1 (% from Q1 Total)	Quarter 2 (% from Q2 Total)	Quarter 3 (% from Q3 Total)	Quarter 4 (% from Q4 Total)	Total Positive Samples
Sputum	67 (16.4)	189 (34.7)	230 (33.0)	170 (32.0)	656 (30.1)
Bronchial aspiration	133 (32.6)	130 (23.9)	132 (19.0)	98 (18.5)	493 (22.6)
Hemoculture	31 (7.6)	28 (5.1)	59 (8.5)	45 (8.5)	163 (7.5)
Urine culture	67 (16.4)	69 (12.7)	82 (11.9)	66 (12.4)	284 (13.0)
Wound secretion	69 (16.9)	93 (17.1)	141 (20.2)	104 (19.6)	407 (18.6)
Catheter	37 (9.1)	20 (3.7)	30 (4.3)	36 (6.8)	123 (5.6)
Others	4 (1.0)	15 (2.8)	22 (3.2)	12 (2.2)	53 (2.4)
**Total**	**408**	**544**	**696**	**531**	**2179**

## Data Availability

The data are encapsulated within the article. Further details can be obtained upon request from either the primary author or the corresponding author. The data are inaccessible to the public due to the patient privacy regulations governing clinical data.

## Supplementary Materials

The following supporting information can be downloaded at: https://www.mdpi.com/article/10.3390/antibiotics13121130/s1, Table S1: Bacterial infections across various sections (units) of hospital. Table S2: Number of isolates and %MDR, %XDR in total. Figure S1: Resistance Trends of Enterobacterales (%R) Across Quarters in 2023. Figure S2: Resistance Trends of Gram-Positive Organisms (%R) Across Quarters in 2023. Figure S3: Resistance Trends of Non-Fermenters (%R) Across Quarters in 2023.
