# Supplementary material for: Unveiling the Dynamics of Antimicrobial Resistance: A Year-Long Surveillance (2023) at the Largest Infectious Disease Profile Hospital in Western Romania
Source: Antibiotics (Basel). 2024 Nov 25;13(12):1130. doi: 10.3390/antibiotics13121130 (PMC11672838; doi:10.3390/antibiotics13121130)
Supplement: Supplementary file 1 [file antibiotics-13-01130-s001.zip › Table S1 - Bacterial infections across various sections (units) of hospital.pdf]

TotalSpecies	Total	ICU	TOR. S.	RMRU	PN 2	PN 1	ID 2	ID 1	Section
	**207** (55.0)	27	1	12	39	55	24	49	*Staph. aureus MSSA*
**376 (17.1)**	**169** (44.9)	48	1	8	26	56	7	23	*Staph. aureus MRSA*
	**54** (42.8)	8	0	1	10	21	1	13	*Staph. epidermidis MSSE*
**126 (5.7)**	**72** (57.1)	28	0	2	7	22	2	11	*Staph. epidermidis MRSE*
	**411** (79.3)	28	1	43	145	140	19	35	*Klebsiella pn. CARBA- ESBL -*
	**77 (**14.8)	28	0	4	8	12	12	13	*Klebsiella pn. CARBA+*
**518 (23.7)**	**30** (5.7)	3	0	0	4	9	4	10	*Klebsiella pn. ESBL+*
	**296 (**81.9)	13	0	28	69	82	30	74	*E. coli ESBL-*
**361 (16.5)**	**65** (18.0)	13	0	1	5	6	15	25	*E. coli ESBL+*
	**80** (98.7)	7	0	4	19	21	8	21	*Proteus spp. ESBL-*
**81 (3.7)**	**1** (1.2)	0	0	0	0	0	1	0	*Proteus spp. ESBL+*
	**65** (64.3)	18	0	4	18	19	1	5	*Acinetobacter CARBA-*
**101 (4.6)**	**36** (35.6)	22	0	0	3	5	2	4	*Acinetobacter CARBA+*
	**277** (82.4)	7	1	31	99	95	13	31	*Pseudomonas CARBA-*
**336 (15.4)**	**59** (17.55)	18	0	4	11	14	4	8	*Pseudomonas CARBA+*
	**20** (34.4)	7	2	0	1	1	2	7	*Enterococcus spp. VRE- HLAR-*
	**4** (6.8)	2	0	0	0	0	0	2	*Enterococcus spp. VRE+*
**58 (2.6)**	**34** (58.6)	14	0	0	3	4	6	7	*Enterococcus spp. HLAR+*
**222 (10.1)**	**222**	32	3	14	57	52	23	41	*Other Bacteria*
**2179**	**2179**	**323**	**9**	**156**	**524**	**614**	**174**	**379**	**Total**

Table S1. Bacterial infections across various sections (units) of hospital

Table’s legend:

Columns:
The columns in the table represent different types of bacteria and their respective resistance profiles. Here's a brief explanation of each column:
Staph. aureus MSSA: Methicillin-Sensitive *Staphylococcus aureus*
Staph. aureus MRSA: Methicillin-Resistant *Staphylococcus aureus*
Staph. epidermidis MSSE: Methicillin-Sensitive *Staphylococcus epidermidis*
Staph. epidermidis MRSE: Methicillin-Resistant *Staphylococcus epidermidis*
Klebsiella pn. CARBA- ESBL -: *Klebsiella pneumoniae*, non-carbapenemase-producing, non-ESBL-producing
Klebsiella pn. CARBA+: *Klebsiella pneumoniae*, carbapenemase-producing
Klebsiella pn. ESBL+: *Klebsiella pneumoniae*, ESBL-producing (Extended-Spectrum Beta-Lactamases)
E. coli ESBL-: *Escherichia coli*, non-ESBL-producing
E. coli ESBL+: *Escherichia coli*, ESBL-producing
Proteus spp. ESBL-: *Proteus* species, non-ESBL-producing
Proteus spp. ESBL+: *Proteus* species, ESBL-producing
Acinetobacter CARBA-: *Acinetobacter* species, non-carbapenemase-producing
Acinetobacter CARBA+: *Acinetobacter* species, carbapenemase-producing
Pseudomonas CARBA-: *Pseudomonas* species, non-carbapenemase-producing
Pseudomonas CARBA+: *Pseudomonas* species, carbapenemase-producing
Enterococcus spp. VRE- HLAR-: *Enterococcu*s species, non-Vancomycin-Resistant, non-High-Level Aminoglycoside Resistant
Enterococcus spp. VRE+: *Enterococcus species*, Vancomycin-Resistant
Enterococcus spp. HLAR+: *Enterococcus* species, High-Level Aminoglycoside Resistant
Other Bacteria: Other types of bacteria not specifically listed in the table
Total: Sum of all infections recorded in each section

Rows:
The rows represent different sections (units) within the healthcare facility where the infections were recorded:

ID 1: Infectious Disease Unit 1
ID 2: Infectious Disease Unit 2
PN 1: Pulmonology Unit 1
PN 2: Pulmonology Unit 2
RMRU: Respiratory Medical Rehabilitation Unit
TOR. S.: Thoracic Surgery Unit
ICU: Intensive Care Unit
TOTAL: The overall total for each bacterial category across all sections
