# Supplementary material for: Unveiling the Dynamics of Antimicrobial Resistance: A Year-Long Surveillance (2023) at the Largest Infectious Disease Profile Hospital in Western Romania
Source: Antibiotics (Basel). 2024 Nov 25;13(12):1130. doi: 10.3390/antibiotics13121130 (PMC11672838; doi:10.3390/antibiotics13121130)
Supplement: Supplementary file 1 [file antibiotics-13-01130-s001.zip › Table S2 - Number of isolates and %MDR, %XDR in total.pdf]

Table S2 - Number of isolates and %MDR, %XDR in total

Bacteria	Number of Isolates	%MDR	%XDR
Escherichia coli	107	35.51	23.68
Klebsiella pneumoniae	166	42.77	47.88
Staphylococcus aureus	169	44.6	0
Staphylococcus epidermidis	54	58.62	0
Enterococcus spp.	58	47.06	0
Pseudomonas aeruginosa	109	22.02	12.84
Acinetobacter spp.	34	88.24	35.29
