# Supplementary figures and images for: Unveiling the Dynamics of Antimicrobial Resistance: A Year-Long Surveillance (2023) at the Largest Infectious Disease Profile Hospital in Western Romania
Source: Antibiotics (Basel). 2024 Nov 25;13(12):1130. doi: 10.3390/antibiotics13121130 (PMC11672838; doi:10.3390/antibiotics13121130)

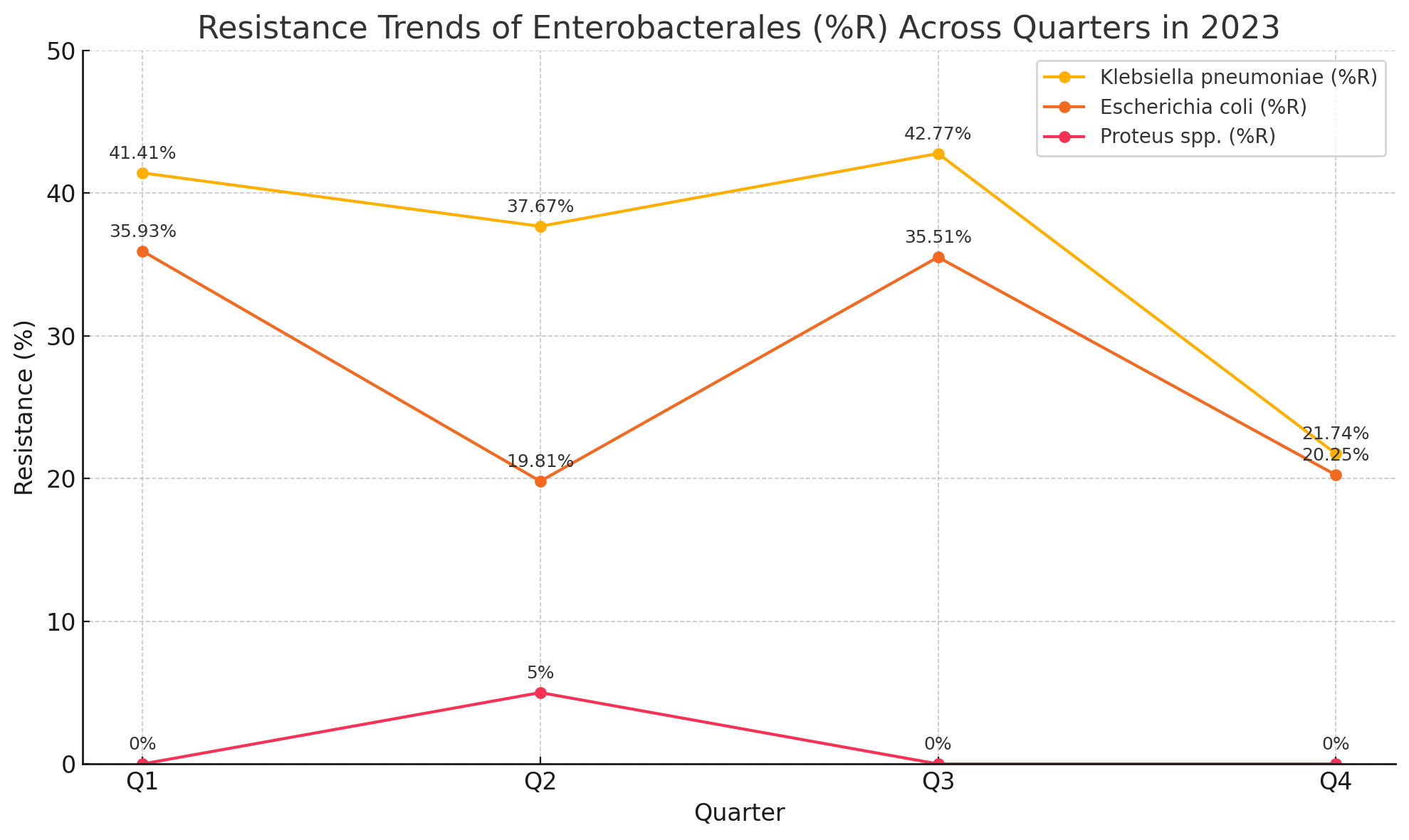

Supplement: Supplementary file 1 [file antibiotics-13-01130-s001.zip › Figure S1 - Quarterly resistance rates (%R) for Enterobacterales.png]

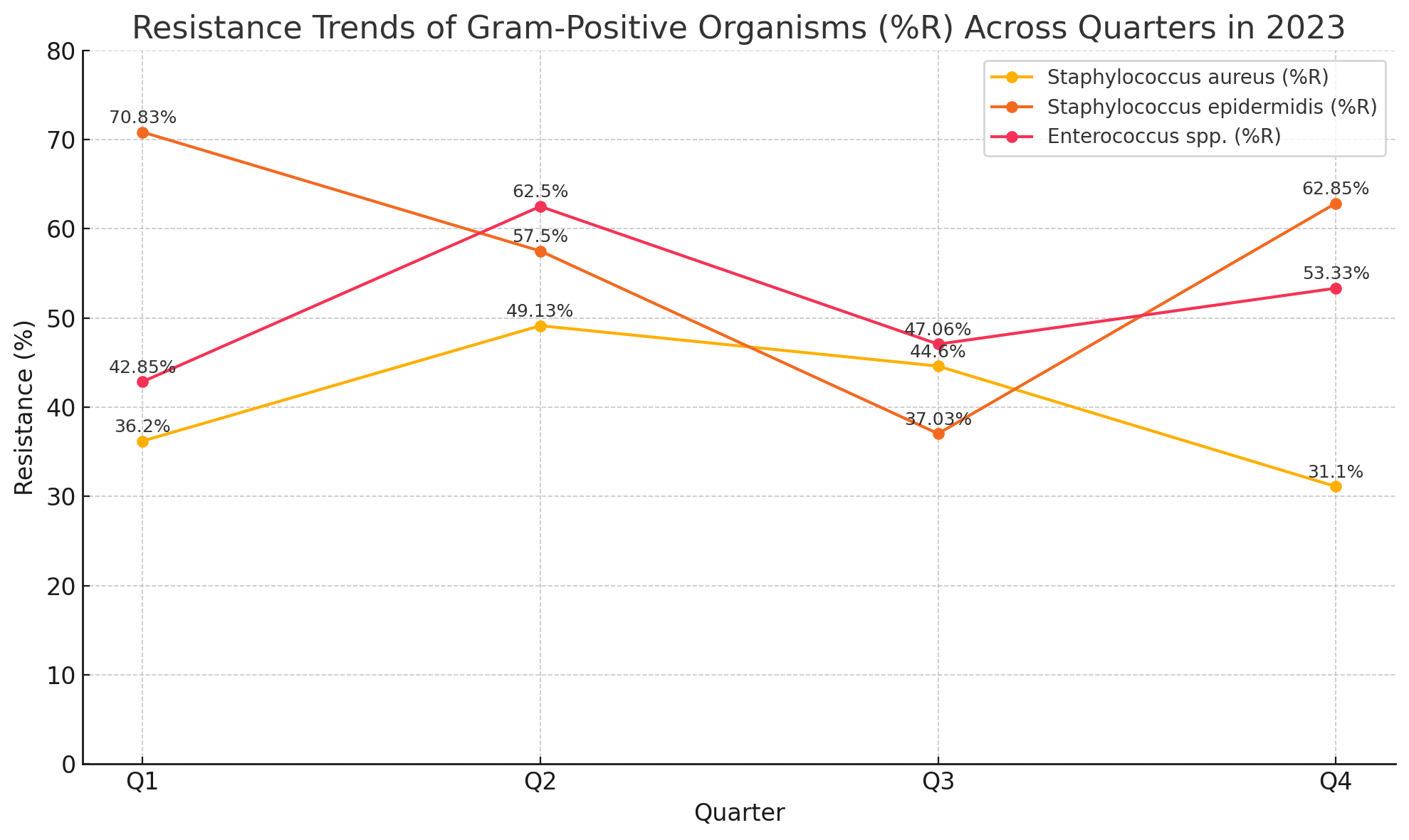

Supplement: Supplementary file 1 [file antibiotics-13-01130-s001.zip › Figure S2 - Quarterly resistance rates (%R) for Gram-positives.png]

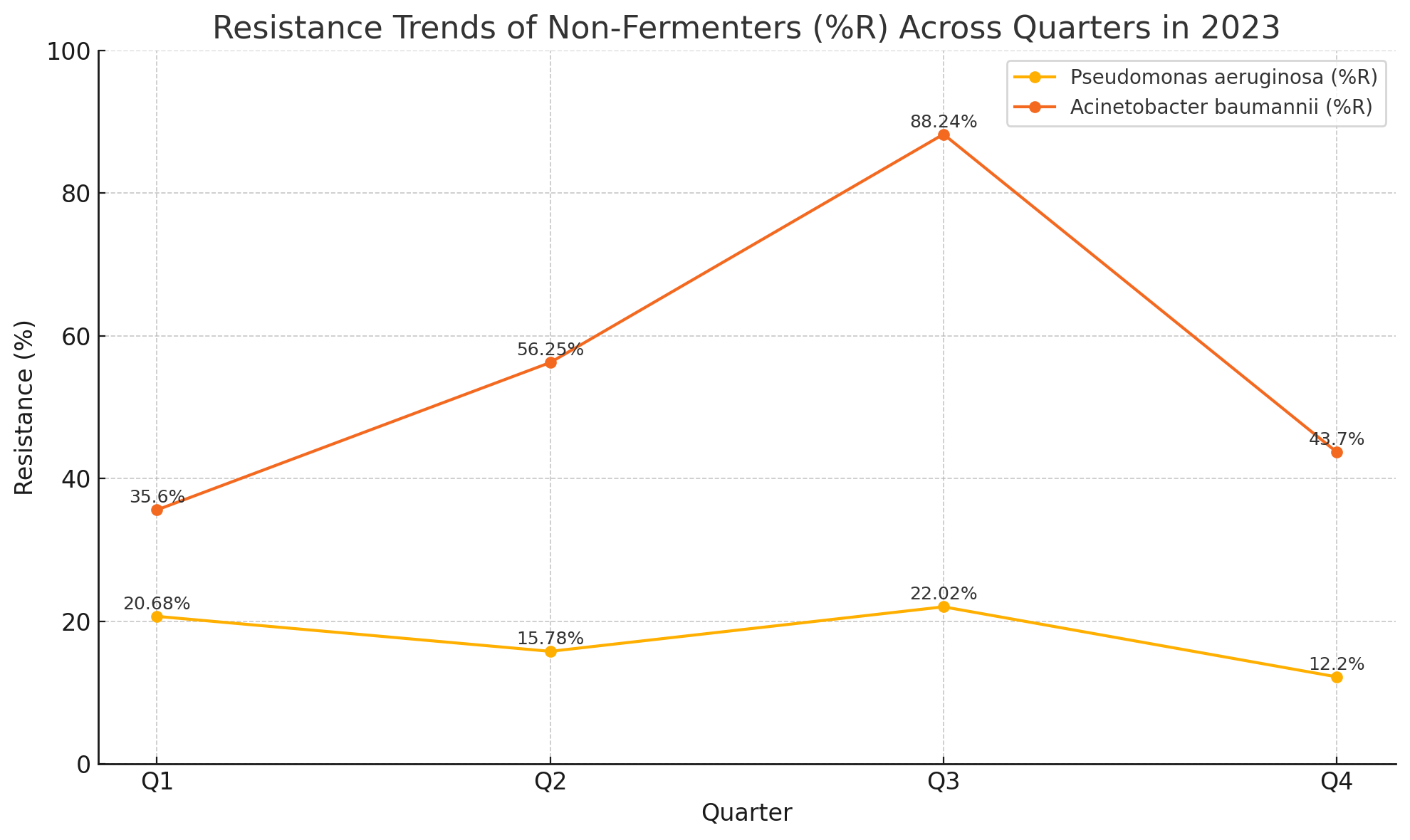

Supplement: Supplementary file 1 [file antibiotics-13-01130-s001.zip › Figure S3 - Quarterly resistance rates (%R) for Non-Fermenters.png]
